# Prevalence and determinants of metabolic syndrome among patients with type 2 diabetes mellitus in the Buea Health District, South West Region, Cameroon

**DOI:** 10.11604/pamj.2026.53.88.46412

**Published:** 2026-02-17

**Authors:** Chu Florence Mbota, Ebot Walter Ojong, Achidi Aduni Ufuan, Ayuk Bertrand Tambe

**Affiliations:** 1 Department of Medical Laboratory Science, Faculty of Health Sciences, University of Buea, Buea, Cameroon,; 2 Department of Biochemistry and Molecular Biology, Faculty of Science, University of Buea, Buea, Cameroon,; 3 Department of Public Health and Hygiene, Faculty of Health Sciences, University of Buea, Buea, Cameroon,; 4 Human Nutrition Division, Faculty of Medicine and Health Sciences, Stellenbosch University, Western Cape, Cape Town, South Africa

**Keywords:** Type 2 diabetes mellitus, metabolic syndrome, glycemic control, Buea, Cameroon

## Abstract

**Introduction:**

metabolic syndrome is a clustering of key cardiovascular risk factors, including abdominal obesity, dyslipidemia, hyperglycemia, and hypertension. Individuals with a combination of metabolic syndrome and type 2 diabetes mellitus (T2DM) have a greater risk of developing microvascular and macrovascular complications, cardiovascular disease, and premature death. This study sought to estimate the prevalence of metabolic syndrome among patients with T2DM in Buea Health District and evaluate its correlation with glycemic control.

**Methods:**

a hospital based cross-sectional study was carried out in the Buea Health District from February to May 2024. One hundred and thirty-nine patients with T2DM were recruited by a systematic sampling. Sociodemographic, lifestyle, and clinical data were collected. Evaluation of anthropometric variables, fasting plasma glucose, triglycerides, high-density lipoprotein cholesterol, blood pressure, and glycated hemoglobin was performed. Data were analyzed using the Statistical Package for Social Sciences. The level of significance was set at p < 0.05.

**Results:**

a total of 139 patients with T2DM were recruited in this study with a mean (SD) age of 59.5 ± 10.95 years. The prevalence of metabolic syndrome was 64.00% and 56.80% using the International Diabetes Federation and National Cholesterol Education Program Adult Treatment Panel criteria, respectively. The prevalence was significantly higher in women (56.81%). Central obesity, hyperglycemia, and hypertension were the most common components of metabolic syndrome, and they were more prevalent in female patients. Patients with T2DM who were females, older, who consumed alcohol, did not practice self-glucose monitoring, and had poor glycemic control were more likely to have metabolic syndrome (p < 0.05).

**Conclusion:**

this study observed a high prevalence of metabolic syndrome among patients with T2DM in Buea, Cameroon. Lifestyle changes and good glycemic control are recommended for the prevention of metabolic syndrome in patients with type 2 diabetes mellitus.

## Introduction

Diabetes mellitus (DM) is a group of metabolic disorders that are characterized by hyperglycemia and insufficiency in insulin production or action [1]. Diabetes mellitus is mostly classified into two major types, namely type 1 (T1DM) and type 2 (T2DM) [2]. In T1DM, there is a deficiency of insulin secretion due to the autoimmune destruction of beta pancreatic cells [3]. The majority (90-95%) of patients with diabetes mellitus have T2DM [4]. Although the exact etiologies of T2DM are not well defined, autoimmune disease, genetic, and environmental factors have been reported to play a major role in its development [5]. A report by the International Diabetes Federation in 2021 revealed that 537 million adults were living with diabetes mellitus [6]. This number is predicted to rise to 643 million by 2030 and 783 million by 2045 [6]. Over 3 in 4 adults with diabetes in 2021 lived in low- and middle-income countries [6]. Diabetes was responsible for 6.7 million deaths in 2021 [6]. In Cameroon, the prevalence of DM is estimated at 5.8% in urban areas, with an estimated 1 million people living with the disease, 70% of whom remain undiagnosed [7]. T2DM and metabolic syndrome (MetS) are closely related public health challenges that have reached alarming epidemic proportions in sub-Saharan Africa.

Metabolic syndrome is characterized by a combination of abdominal obesity, high blood pressure, hyperglycaemia, and dyslipidaemia and poses a significant risk to individuals worldwide [8,9]. Globally, the prevalence of MetS is rising rapidly, and it is highly prevalent in patients with T2DM [10,11]. It is estimated that 20% to 25% of the adult general population and 70% to 80% of T2DM patients had MetS worldwide [12]. Individuals with T2DM have a greater risk of developing metabolic syndrome compared to healthy individuals [13]. In Cameroon, there is a paucity of data on the determinants of MetS in the diabetic population. Furthermore, few studies have been carried out to investigate gender differences in the prevalence of metabolic syndrome components in the Cameroonian diabetic population. Therefore, the current study aimed to determine the prevalence and determinants of MetS among patients with T2DM in South Western Cameroon using two criteria: the International Diabetes Federation (IDF) criteria and the 2004 National Cholesterol Education Program-Adult Treatment Panel (NCEP-ATP III 2004) criteria. This will enhance understanding of MetS within this vulnerable population and provide valuable information for healthcare interventions.

## Methods

**Study design and location:** this was an analytical cross-sectional study of the determinants of MetS among patients with T2DM conducted in two diabetic centres in Fako Division, South West Region, Cameroon. The participating facilities were the diabetic clinic of the Buea Regional Hospital and the Muea Sub-Divisional Health Centre. These facilities cater to the health needs of urban and semi-urban communities in the South West Region of Cameroon. A total of one hundred and thirty-nine (139) participants were recruited from both sites from February to May, 2024. One hundred and ten (110) participants were recruited from the Buea Regional Hospital (BRH), while twenty-nine (29) participants were recruited from the Sub-Divisional Health Centre (CMA), Muea.

**Study participants:** consenting adult patients (aged 21 years and above) with a confirmed diagnosis of type 2 diabetes mellitus using the World Health Organization Criteria who were on an overnight fast for 8 to 12 hours were included in the study. Eight (8) to 12 hours of overnight fasting was taken as a criterion because it indicates insulin sensitivity, assessment of basal insulin, and glucose stability [14]. Pregnant women, hypertensive patients, patients with gestational diabetes mellitus, patients with incomplete data, and T2DM patients who were not on an overnight fast for 8 to 12 hours were excluded.

**Sample size determination:** the sample size was estimated using a single population formula [15]:

$$n=\frac{z^{2}\left(p\right)\left(1-p\right)}{e^{2}}$$

Where n = sample size, z= critical value at 95% confidence level usually set at 1.96, p=prevalence (5.8% prevalence rate of DM in Cameroon) [16], q = 1-p, d= imprecision of 5%. Inputting the variables:

n=[((1.96)^2^(0.058)(1-0.058))/(0.05)^2^]=84

However, a total sample size of 139 was studied.

**Sampling technique:** the Buea Health District and the two selected hospitals were selected by purposive sampling. The Buea Regional Hospital and the Muea Sub-Divisional Health Centre are situated in the South-West Region of Cameroon. They serve as primary health care facilities and cater for diverse population. Participants who met the inclusion criteria were selected by systematic sampling. Starting from the first among the 700 registered patients, every fifth patient was selected, and a sample size of 139 was attained.

**Data collection:** the dependent variable is the presence of metabolic syndrome. The independent variables are sociodemographic, biochemical, clinical, and lifestyle data. The clinician attending to the patient confirmed that the patient had supper as their last meal. After obtaining written consent, participants were interviewed face-to-face by the research team using a structured questionnaire to collect the necessary sociodemographic (gender, age, marital status, religion, education, employment status, area of residence), lifestyle (smoking status, physical activity, alcohol consumption status), and biochemical data. Clinical data (family history of diabetes, duration of diabetes, type of diabetes treatment, glucose self-monitoring, and BMI) were extracted from participants´ medical files. A trained nurse measured participants´ height with them barefoot using a portable stadiometer and recorded them to the nearest 0.1 cm. Participants´ body weight was measured with them wearing light clothing, using a calibrated scale, and recorded to the nearest 0.1 kg. Body mass index (BMI) was calculated using the formula: weight (kg) divided by the square of height (m^2^). Waist circumference (WC) was measured using a measuring tape and recorded to the nearest 0.1 cm. Systolic and diastolic blood pressures (BP) were measured three times using a mercury sphygmomanometer following a 10-minute rest, and the average of the readings was recorded. About 5 mL of venous blood was drawn from each participant by a trained laboratory scientist. About 3 mL was dispensed into a labelled ethylene diamine tetra-acetic acid (EDTA) tube, processed, and used for the measurement of high-density lipoprotein (HDL-c) and triglycerides (TG) concentration. The remaining was dispensed into a fluoride oxalate tube for fasting plasma glucose (FPG) measurement. FPG, HDL-c, and TG were measured by enzymatic colorimetric methods using the CYANSmart semi-automated chemistry analyzer (Cypress Diagnostics, ISO 13485-2016, Belgium). Glycated haemoglobin (HbA1c) was measured by an ion exchange high-performance liquid chromatography method.

**Operational definitions:** the International Diabetes Federation (IDF) criteria and the NCEP-ATP III criteria were used for the diagnosis of metabolic syndrome. According to IDF, metabolic syndrome is defined as central obesity (defined by waist circumference) plus any two of the four risk factors: raised triglycerides, low HDL-c, raised blood pressure, and/or raised FPG level [17]. According to the NCEP-ATP III criteria, metabolic syndrome is present when at least any three or more of the five criteria is present; waist circumference (WC) ≥ 102 cm (M), ≥ 88 cm (F), FG ≥ 105 mg/dL, TG ≥ 150 mg/dL HDL< 40 mg/dL (M), < 50 mg/dL (F), hyperglycemia ≥ 130 mmHg systolic and/or ≥ 84 mmHg diastolic [17].

Overweight was defined as a BMI of 25-29 kg/m^2^ and obesity was defined as BMI ≥ 30 kg/m^2^ according to WHO guidelines [18]. American Heart Association guidelines were used to define hypertension as BP ≥ 140/90 mmHg [19]. Central obesity was considered in males with a WC > 102 cm and females with a WC > 88 cm according to the National Heart, Lung, and Blood Institute recommendations [20]. T2DM was defined based on WHO criteria (fasting plasma glucose values ≥ 7.0 mmol/L [126 mg/dL], 2-hour post-load plasma glucose ≥ 11.1 mmol/L [200 mg/dL] [21], glycated hemoglobin [HbA1c] ≥ 6.5% [48 mmol/mol]; or random blood glucose ≥ 11.1 mmol/L [200 mg/dL]) in the presence of signs and symptoms considered related to diabetes [21]. Good glycemic control was considered when HbA1c < 7 and poor glycemic control when HbA1c > 7 according to WHO recommendations [22].

**Ethical consideration:** ethical approval for this study was obtained from the Institutional Review Board of the Faculty of Health Sciences, University of Buea, Buea, Cameroon (Reference No: 2024/2287-01/UB/SG/IRB/FHS). Authorization to collect research data was obtained from each hospital. Participation in this study was voluntary, and written informed consent was obtained from each participant before the commencement of the study. Data obtained were treated as confidential and used only for the purpose of the study.

**Statistical analyses:** data were entered into a Microsoft Excel Spreadsheet (IBM, USA) and statistical analyses were conducted using the Statistical Package for the Social Sciences software version 26 (Chicago, IL, USA). Categorical variables are presented as frequencies and percentages, while numerical variables with symmetrical distribution are expressed as means ± standard. The prevalence of metabolic syndrome using each criterion was determined by the number of affected patients over the total number of patients and presented as frequency (percentage (%)). The Pearson's chi-square (χ^2^) test was used to compare the prevalence of metabolic syndrome using the two criteria. Sociodemographic, clinical, and lifestyle factors associated with metabolic syndrome were analyzed using univariate and multivariate analysis in the study population. Variables with a p-value of less than 0.2 in the univariable analysis were included in the multivariable model. The results are presented as the odds ratio with the corresponding 95% confidence interval. The Student t-test was used to compare the means of measured anthropometric and biochemical parameters of patients with metabolic syndrome and those without metabolic syndrome. For all tests, statistical significance was set at a p < 0.05.

## Results

**Sociodemographic characteristics of participants:** overall, 139 T2DM patients participated in this study. One hundred and ten (110) participants were recruited from the Regional Hospital Buea and 29 from the Sub-Divisional Hospital Muea-Buea. The majority, 115 (82.7%) of the participants were females, and only 24 (17.3%) participants were males. The mean (SD) age of participants was 59.5 ± 10.95 years, with about two-thirds of them 85 (61.2%) between ages 50-70. More than half of the participants were married, 76 (54.7%), and about two-thirds, 84 (60.4%) of them lived in urban areas.

**Lifestyle and clinical parameters of study participants:** one-third of the participants were reported to have T2DM for a duration of 5-10 years 46 (33.1%). About half 69 (49.6%) of the participants had a first-degree relative with a family history of T2DM. Less than half of the participants were on metformin 66 (47.5%) as compared to other antidiabetic medications. The majority of participants, 121 (87.1%), reported practicing glucose self-monitoring. Also, the majority, 136 (97.8%) of participants were non-smokers, and 95 (68.3%) were physically active. Furthermore, 85 (61.2%) of the participants did not consume alcohol. Lastly, most 102 (73.4%) of participants consumed fatty food.

**Prevalence of metabolic syndrome in patients with type 2 diabetes mellitus:** the prevalence of metabolic syndrome using the IDF criteria of 64.0% was significantly higher than the prevalence based on the NCEP-ATP III criteria of 56.80% (χ^2^= 102.8, p < 0.001).

**Prevalence of metabolic syndrome based on gender using IDF and NCEP criteria:** more female participants had MetS compared to male participants. The prevalence of metabolic syndrome in females using the IDF criteria was 50.0% compared to 6.67% in males. The prevalence of metabolic syndrome in females using the NCEP-ATP III criteria was 51.90% compared to 3.79% in males.

**Prevalence of metabolic syndrome components among male and female participants using the IDF criteria:** central obesity was more prevalent in female patients with T2DM compared to males (p=0.004) (Table 1).

**Table 1:** prevalence of metabolic syndrome components among male and female participants using the International Diabetes Federation criteria

Parameters (cut-offs)	Male (n=24) n (%)	Female (n=115) n (%)	χ^2^	p-value
Central obesity (WC≥94 cm (M), ≥80 cm(F), cm)	16 (11.51)	103 (74.09)	8.45	0.004
Hyperglycemia (FPG≥100mg/dL)	10 (4.77)	67 (31.93)	2.21	0.14
Dyslipidemia: TG (≥150mg/dL)	6 (4.32)	45 (32.38)	1.71	0.19
Dyslipidemia: HDL-cholesterol (<40mg/dL (M),<50mg/dL (F)	8 (5.75)	41 (29.45)	0.05	0.83
Hypertension: systolic BP (≥130mmHg)	6 (4.31)	53 (38.09)	3.61	0.06
Hypertension: diastolic BP (>85mmHg)	6 (4.32)	28 (20.18)	0.005	0.95
BMI (Kg/m^2^): underweight (<18.5)	0 (0.00)	1 (0.70)	0.21	0.65
BMI: normal weight (18.5-24.9)	1 (0.72)	8 (5.75)	0.26	0.61
BMI: overweight (25.0-29.9)	4 (2.88)	32 (3.02)	1.29	0.26
BMI: obese (≥30)	5 (3.59)	37 (26.60)	1.21	0.27

WC: waist circumference; BP: blood pressure; M: male; F: female; FPG: fasting plasma glucose; TG: triglycerides; HDL: high-density lipoprotein; BMI: body mass index

**Prevalence of metabolic syndrome components among male and female participants using the NCEP criteria:** central obesity, hyperglycemia, and hypertension were more prevalent in female patients with T2DM compared to males (p < 0.05) (Table 2).

**Table 2:** prevalence of metabolic syndrome components among male and female participants using the National Cholesterol Education Program criteria

Parameters (cut-offs)	Male (n=24) n (%)	Female (n=115) n (%)	χ^2^	p-value
Central obesity (WC≥102 cm (M), ≥88 cm(F))	9 (6.47)	95 (68.33)	21.45	<0.001
Hyperglycemia (FPG≥100mg/dL)	6 (4.31)	63 (45.29)	7.05	0.008
Dyslipidemia: TG (≥150mg/dL)	4 (2.88)	41 (29.52)	3.27	0.07
Dyslipidemia: HDL-cholesterol (<40mg/dL (M),<50mg/dL (F)	4 (2.88)	36 (25.92)	2.08	0.15
Hypertension: systolic BP (≥130mmHg)	4 (2.88)	49 (35.22)	5.66	0.02
Hypertension: diastolic BP (>85mmHg)	4 (2.88)	25 (18.02)	0.31	0.58
BMI (Kg/m^2^): underweight (<18.5)	0 (0.00)	0 (0.00)	-	-
BMI: normal weight (18.5-24.9)	0 (0.00)	7 (5.04)	1.54	0.22
BMI: overweight (25.0-29.9)	2 (1.44)	28 (20.16)	3.00	0.08
BMI: obese (≥30)	4 (2.88)	37 (26.62)	2.29	0.13
MetS based on ATP III	3 (3.79)	41 (51.90)	4.37	0.01
MetS based on IDF	6 (6.67)	45 (50.0)	3.98	0.004

WC: waist circumference; BP: blood pressure; M: male; F: female; FPG: fasting plasma glucose; TG: triglycerides; HDL: high-density lipoprotein; BMI: body mass index

### Factors associated with metabolic syndrome in patients with type 2 diabetes mellitus in the Buea Health District

**Sociodemographic factors associated with metabolic syndrome:** Table 3 and Table 4 show significant associations between MetS, gender, and age using both the IDF and NCEP-ATP III criteria. Based on the IDF criteria, male participants were less likely to have metabolic syndrome compared to females (odds ratio (OR) = 0.33; 95% confidence interval (Cl): 0.13-0.80, p = 0.02). Also, age was significantly associated with metabolic syndrome. Participants aged >70 years were 4 times more likely to have MetS compared to younger participants aged 30 - 50 years (OR=3.50; 95% CI =1.11-11.07; p =0.04). Based on the NCEP-ATP III criteria, males were less likely to have metabolic syndrome compared to females (OR = 0.19; 95% CI =0.07-0.52, p = 0.001) (Table 4).

**Table 3:** sociodemographic factors associated with metabolic syndrome using the International Diabetes Federation criteria

Factors	Metabolic syndrome n (%)		Metabolic syndrome	
			cOR (95% CI)	p-value
	No	Yes		
**Sex**				
Female	36 (31.3)	79 (68.7)	1	
Male	14 (58.3)	10 (41.7)	0.33 (0.13-0.80)	0.02
**Age groups (years)**				
30-50	14 (53.8)	12 (46.2)	1	
51-70	29 (34.1)	56 (65.9)	2.25 (0.92-5.49)	0.11
>70	7 (25.0)	21 (75.0)	3.50 (1.11-11.07)	0.04
**Employment status**				
Employed	5 (29.4)	12 (70.6)	1	
Self employed	26 (43.3)	34 (56.7)	3.74 (0.73-19.05)	0.18
Unemployed	19 (30.6)	43 (69.4)	1.16 (0.54-2.48)	0.70
**Marital status**				
Married	28 (36.8)	48 (63.2)	1	
Single	13 (52.0)	12 (48.0)	0.54 (0.17-1.74)	0.18
Widowed	9 (23.7)	29 (76.3)	1.88 (0.78-4.54)	0.70
**Place of residence**				
Rural	17 (40.5)	25 (59.5)	1	
Semi-urban	2 (15.4)	11 (84.6)	3.75 (0.43-32.52)	0.18
Urban	31 (36.9)	53 (63.1)	0.83 (0.35-1.95)	0.70

**Table 4:** sociodemographic factors associated with metabolic syndrome among patients with type 2 diabetes mellitus using the National Cholesterol Education Program-Adult Treatment Panel III criteria

Factors	Metabolic syndrome n (%)		Metabolic syndrome	
			cOR (95% CI)	p-value
	No	Yes		
**Sex**				
Female	42 (36.52)	73 (63.48)	1	
Male	18 (75)	6 (25)	0.19 (0.07-0.52)	0.001
**Age group (years)**				
30-50	16 (61.53)	10 (38.46)	1	
51-70	36 (42.35)	49(57.65)	2.18(0.89-5.35)	0.12
>70	8 (28.57)	20 (71.43)	3.50 (1.28-12.49)	0.03
**Work status**				
Employed	7 (41.17)	10 (58.83)	1	
Self employed	29 (48.34)	31 (51.66)	0.75 (0.25-2.23)	0.78
Unemployed	24 (38.71)	38 (61.29)	1.11 (0.37-3.30)	1.00
**Marital status**				
Married	33 (43.43)	3 (56.57)	1	
Single	15 (60)	10 (40)	0.51 (0.20-1.28)	0.17
Widowed	12 (31.58)	26 (48.42)	1.66 (0.73-3.78)	0.31
**Residence**				
Rural	19 (45.24)	23 (54.76)	1	
Semi-urban	5 (38.46)	8 (61.54)	1.32 (0.37-4.72)	0.76
Urban	36 (42.86)	48 (57.14)	0.10 (0.52-2.32)	0.85

**Clinical and lifestyle characteristics associated with metabolic syndrome using IDF criteria:** based on the IDF criteria, alcohol consumption was significantly associated with metabolic syndrome. Participants who consumed alcohol were two times more likely to have metabolic syndrome compared to non-alcoholics (OR = 2.37; 95% CI = 0.21-3.02, p = 0.007) (Table 5).

**Table 5:** clinical and lifestyle characteristics associated with metabolic syndrome using the International Diabetes Federation criteria

Factors	Metabolic syndrome n (%)		Crude	
			cOR (95% CI)	p-value
	No	Yes		
**Diabetes duration (years)**				
<1	10 (38.5)	16 (61.5)	1	
1-5	15 (38.5)	24 (61.5)	1.94 (0.31-2.85)	1.00
6-10	17 (37.0)	29 (63.0)	1.51 (0.48-4.69)	1.00
>10	8 (28.6)	20 (71.4)	1.10 (0.33-3.73)	0.57
**Diabetes medication**				
Metformin	24 (36.4)	42 (63.6)	1	
Diapride	4 (36.4)	7 (63.6)	0.47 (0.12-1.84)	0.27
Insulin	11 (34.4)	21 (65.6)	0.96 (0.34-2.68)	1.00
Glimepiride	4 (57.1)	3 (42.9)	0.36 (0.07-1.79)	0.34
Others	7 (30.4)	16 (69.6)	0.76 (0.25-2.29)	0.77
**Family history of T2DM**				
No	20 (31.7)	43 (68.3)	1	
First line relatives	28 (40.6)	41 (59.4)	0.68 (0.32-1.54)	0.37
Second line relatives	2 (28.6)	5 (71.4)	1.16 (0.21-6.51)	1.00
**Smoking**				
No	48 (35.3)	88 (64.7)	1	
Yes	2 (66.7)	1 (33.3)	0.27 (0.06-7.29)	0.29
**Physical activity**				
No	17 (38.6)	27 (61.4)	1	
Yes	33 (34.7)	62 (65.3)	0.94 (0.40-2.19)	0.25
**Alcohol consumption**				
No	23 (27.1)	62 (72.9)	1	
Yes	27 (50.0)	27 (50.0)	2.37 (0.21-3.02)	0.007
**Eating fatty diet (fried chicken, butter)**				
No	11 (26.2)	31 (73.8)	1	
Yes	32 (35.5)	58 (64.4)	0.81 (0.33-1.98)	0.82
**Self-glucose monitoring**				
No	10 (55.6)	8 (44.4)	1	
Yes	40 (33.1)	81 (66.9)	0.53 (0.40-3.71)	0.07
**BMI**				
Normal weight	10 (50.0)	10 (50.0)	1	
Under weight	2 (66.7)	1 (33.3)	0.50 (0.04-6.44)	1.00
Overweight	23 (39.0)	36 (61.0)	1.57 (0.56-4.34)	0.44
Obese	15 (26.3)	42 (73.7)	2.80 (0.97-8.05)	0.09

**Clinical and lifestyle characteristics associated with metabolic syndrome in patients with T2DM using NCEP-ATP III criteria:** based on the NCEP-ATP III criteria, glucose self-monitoring, alcohol consumption, and body mass index were significantly associated with metabolic syndrome. Participants who consumed alcohol were two times more likely to have metabolic syndrome compared to non-alcoholics (cOR=2.34, 95% Cl 0.17-4.68). Also, participants who practiced self-glucose monitoring were three times less likely to have MetS compared to those who did not practice self-glucose monitoring (cOR = 3.04; 95% CI =1.07-8.65, p =0.04). Finally, obese participants were four times more likely to have MetS compared to participants with normal weighted participants (cOR = 3.84; 95% CI = 1.33-11.15, p = 0.02) (Table 6).

**Table 6:** Clinical and lifestyle characteristics associated with metabolic syndrome using the National Cholesterol Education Program-Adult Treatment Panel III criteria

Factors	Metabolic syndrome n (%)		Crude	
			cOR (95% CI)	p-value
	No	Yes		
**Diabetes duration (years)**				
<1	11 (42.3)	15 (57.7)	1	
1-5	17 (43.6)	22 (56.4)	0.95 (0.35-2.59)	1.00
6-10	22 (47.8)	24 (52.2)	0.80 (0.30-2.11)	0.80
>10	10 (35.7)	18 (64.3)	1.32 (0.44-3.95)	0.78
**Diabetes medication**				
Metformin	29 (43.9)	37 (56.1)	1	
Diapride	4 (36.4)	7 (63.6)	1.37 (0.37-5.14)	0.75
Insulin	15 (46.9)	17 (53.1)	0.89 (0.38-2.07)	0.83
Glimepiride	4 (57.1)	3 (42.9)	0.59 (0.12-2.83)	0.69
Others	8 (37.8)	15 (65.2)	1.47 (0.55-3.94)	0.47
**Family history of T2DM**				
No	25 (39.7)	38 (60.3)	1	
First line relatives	33 (47.8)	36 (52,2)	0.72 (0.36-1.43)	0.38
Second line relatives	2 (28.6)	5 (71.4)	1.64 (0.29-9.15)	0.69
**Smoking**				
No	48 (35.3)	88 (64.7)	1	
Yes	2 (66.7)	1 (33.3)	0.27 (0.06-7.29)	0.29
**Physical activity**				
No	20 (45.4)	24 (54.6)	1	
Yes	40 (42.1)	55 (57.9)	1.12 (0.54-2.31)	0.45
**Alcohol consumption**				
No	28 (32.9)	57 (67.1)	1	
Yes	32 (59.3)	22 (40.7)	2.34 (0.17-4.68)	0.003
**Eating a fatty diet (fried chicken, butter)**				
No	15 (40.5)	22 (59.5)	1	
Yes	45 (44.1)	57 (55.9)	0.86 (0.40-1.85)	0.85
**Self-glucose monitoring**				
No	12 (66.7)	6 (33.3)	1	
Yes	48 (39.7)	73 (60.3)	3.04 (1.07-8.65)	0.04
**BMI**				
Normal weight	12 (60.0)	8 (40.0)	1	
Under weight	3 (100)	-	-	0.53
Overweight	29 (49.1)	30 (50.9)	1.55 (0.55-4.35)	0.45
Obese	16 (28.1)	41 (71.9)	3.84 (1.33-11.15)	0.02

**Association between metabolic syndrome and glycemic control:** patients with T2DM with good glycemic control were less likely to have metabolic syndrome compared to those with poor control (Table 7).

**Table 7:** association between metabolic syndrome and glycemic control

Factors	Metabolic syndrome n (%)		Metabolic syndrome	
			OR (95% CI)	p-value
	No	Yes		
**Glycated haemoglobin (HbA1c)**				
Poor	27 (27.6)	71 (72.4)	1	
Good	23 (56.1)	18 (43.9)	0.29 (0.14-0.64)	0.002
**Fasting blood sugar (FBS)**				
Poor	32 (40.0)	48 (60.0)	1	
Good	18 (30.5)	41 (69.5)	1.52 (0.74-3.09)	0.29

## Discussion

Metabolic syndrome is one of the serious public health problems among patients with type 2 diabetes mellitus. A systematic review and meta-analysis reported that almost two out of three patients with type 2 diabetes mellitus in sub-Saharan African countries have metabolic syndrome [23].

This study assessed the prevalence and identified factors associated with metabolic syndrome among patients with type 2 diabetes mellitus in the Buea Health District. The findings of this study indicate that the prevalence of MetS was 64.0% and 56.8% using IDF and NCEP-ATP criteria, respectively. The prevalence was slightly higher when the IDF criteria were used compared to the NCEP-ATP III 2004 criteria. Disparities in the prevalence of MetS defined using different criteria have been reported [24]. These differences can be attributed to variations in the diagnostic components and cut-offs used by each definition as reported by many studies in different countries [23,24].

The prevalence of metabolic syndrome of 64.0% reported in the present study using the IDF definition is slightly higher than the weighted pooled prevalence of MetS of 60.8% (95% CI: 50.7-70.0) reported by a recent systematic review in sub-Saharan Africa using the same criteria [24]. However, it is similar to the prevalence of 63.9% reported by Unadike *et al*. in Nigeria [25]. Furthermore, the prevalence of metabolic syndrome of 56.8% reported in the present study using the NCEP-ATP III definition is lower than the weighted pooled prevalence of 63.1% (95% CI: 57.9-68.1) reported by the recent systematic review in sub-Saharan Africa using the same criteria [24].

This prevalence is also lower than the prevalence of 41.3 reported by Charkos *et al*. in Ethiopia [26] and Osei-Yeboah *et al*. in Ghana [27]. The prevalence of metabolic syndrome in the present study reported by both criteria is lower than previous reports by Kengne *et al*. in Cameroon [28]. The differences in the prevalence of metabolic syndrome in different countries can also be attributed to differences in lifestyle factors, diet, study design, and healthcare systems [29].

The present study also found that using both the NCEP-ATP III and IDF criteria, the prevalence of metabolic syndrome was higher in females compared to males. The pathogenesis of cardio-metabolic risk factors reported in women is different from men. These observed differences are due to sex differences in body fat distribution and insulin resistance. The differences are also partly due to the effects of sex hormones and glucose. A study carried out by Ogbera *et al*. reported that the proportion of males with the MetS was comparable to that of females [30].

In the present study, the prevalence of metabolic syndrome components was evaluated, and it was found that central obesity was the most prevalent component, followed by hyperglycaemia and hypertension. Visceral adiposity is known to play a central role in insulin resistance, which results in hyperglycaemia and a heightened risk of type 2 diabetes mellitus and cardiovascular diseases [31]. Several studies have reported central obesity, hyperglycaemia, hypertension, and dyslipidaemia as predominant components of the metabolic syndrome [21].

The present study further reveals that central obesity, hyperglycaemia, and hypertension were more prevalent in female patients with T2DM compared to males. A study by Ogbera *et al*. revealed that the prevalence of central obesity was higher in females compared to males [30]. Central obesity develops before other metabolic syndrome components and has been reported to play a key role in its development [32]. Although the role of central obesity in patients with the metabolic syndrome is not fully understood, active brown adipocytes have been found to be metabolically active. In addition, several studies have reported a correlation of central obesity with hypertension, insulin resistance, and dyslipidemia [33]. Shera *et al*. carried out a large population study and reported a higher prevalence of obesity and hypertension in female patients with diabetes mellitus compared to males [34]. Several studies have reported gender differences in the prevalence of metabolic syndrome components [35]. However, the findings are inconsistent and may be attributed to confounding factors like age, genetic traits, lifestyle factors, and socioeconomic status [36].

Lastly, the present study also evaluated the sociodemographic, lifestyle, and clinical predictors of the metabolic syndrome among patients with type 2 diabetes mellitus.

Our findings reveal that using both the IDF and NCEP-ATP III criteria, male patients with T2DM were less likely to have metabolic syndrome compared to females. This finding is consistent with reports from previous studies conducted in Ghana [37], Bangladesh [38], and mainland China [39] and sub-Saharan Africa [40]. Gender-specific factors, including increased waist circumference, menopause, increased body weight, contraceptive use, and elevated body weight, explain why females have a higher risk of metabolic syndrome compared to males [41]. Findings from this study further reveal that aging increases the risk of metabolic syndrome among type 2 diabetes mellitus patients. Physiological changes that occur with age affect metabolic regulation and contribute to the increased risk of MetS. Also, findings from the present study reveal that participants who had consumed alcohol were 2 times more likely to have MetS compared to those who did not consume alcohol. Shita *et al*. have also reported significant differences in the prevalence of metabolic syndrome among patients with T2DM who were alcoholics and those who were non-alcoholics in Ethiopia [42]. This could be because alcohol consumption in diabetics can worsen blood sugar control, cause disturbances in fat metabolism, and inhibit fat oxidation, which can lead to increased adiposity [43]. Excessive alcohol consumption can also lead to pro-inflammatory changes that can increase the risk of atherosclerotic cardiovascular disease [43].

## Conclusion

This study reports a higher prevalence of metabolic syndrome among patients with type 2 diabetes mellitus using the IDF criteria (64.0%) compared to the NCEP-ATP III criteria (56.8%). The prevalence of metabolic syndrome was higher in females than in males. Central obesity, hyperglycaemia, and hypertension are the most common components of metabolic syndrome in patients with type 2 diabetes mellitus and were more prevalent in females. Older age, female gender, obesity, alcohol consumption, and non-glucose self-monitoring are associated with higher risks of metabolic syndrome in type 2 diabetes mellitus patients in South Western Cameroon. Findings from this study underscore the need for a holistic approach in type 2 diabetes management, which targets the metabolic syndrome component. Patients with type 2 diabetes mellitus should be encouraged to adopt healthy lifestyles and undertake regular screening for hypertension and obesity in order to prevent metabolic syndrome and delay the onset of cardiovascular disease.

### What is known about this topic

- Among studies done on the prevalence and components of metabolic syndrome among patients with type 2 diabetes mellitus in Africa, few studies have evaluated the prevalence using both the NCEP-ATP III 2004 and IDF criteria;
- Previous studies in Africa have reported the prevalence of MetS components based on either the NCEP-ATP III 2004 criteria or the IDF criteria, but not both;
- There is a paucity of data on gender differences in the prevalence of metabolic syndrome in Cameroon, and previous studies have not associated metabolic syndrome with glycemic control.

### What this study adds

- This study adds to the few studies that have evaluated the prevalence of metabolic syndrome using both the NCEP-ATP III 2004 and IDF criteria;
- Additionally, this study reports the prevalence of metabolic syndrome components based on both the NCEP-ATP III 2004 criteria and IDF criteria and compares prevalence among male and female patients with type 2 diabetes mellitus;
- It also evaluates the association of metabolic syndrome and glycemic control in patients with type 2 diabetes mellitus in Cameroon.
